# Assessment of alarm fatigue among intensive care unit nurses: a cross-sectional study

**DOI:** 10.1186/s12912-025-03781-8

**Published:** 2025-09-02

**Authors:** Anja Hohenwallner, Marina Ufelmann, Andrea Ellermeyer, Anna Scholze, Laura Borgstedt, Akira-Sebastian Poncette, Kristina Fuest

**Affiliations:** 1 Department of Anaesthesiology & Intensive Care Medicine, TUM University Hospital Rechts der Isar, Ismaninger Straße 22, 81675 Munich, Germany; 2 Directorate of Nursing - Department of Nursing Science, TUM University Hospital Rechts der Isar, Munich, Germany; 3 Department of Anesthesiology and Intensive Care Medicine (CCM/CVK), Charité - Universitätsmedizin Berlin, Corporate Member of Freie Universität Berlin and Humboldt-Universität Zu Berlin, Berlin, Germany; 4 Institute of Medical Informatics, Charité - Universitätsmedizin Berlin, Corporate Member of Freie Universität Berlin and Humboldt-Universität Zu Berlin, Berlin, Germany

**Keywords:** Alarm fatigue, Intensive care unit, Clinical alarms, Critical care nurse, Patient safety

## Abstract

**Background:**

Alarm fatigue occurs when ICU personnel are exposed to an excessive number of alarms, resulting in desensitization, improper alarm management, and reduced patient safety. This study aimed to assess the extent of alarm fatigue among ICU nurses at a German university hospital using the Charité Alarm Fatigue Questionnaire (CAFQa) and to examine differences based on working hours and professional experience.

**Methods:**

An observational cross-sectional study design was used. Data were collected via an online survey from nurses working in five intensive care units (ICUs) of a German university hospital between January and March 2024. The questionnaire comprised 27 items based on the Charité Alarm Fatigue Questionnaire (CAFQa). Additionally, participants rated their perceived alarm fatigue on a visual analogue scale ranging from 0 to 100%. Above that, participants were asked to provide information regarding their ICU experience and monthly workdays. Statistical significance was assessed using two-sample t-tests. Correlations between CAFQa scores and self-reported alarm fatigue were analysed using Pearson’s correlation coefficient. A p-value of < 0.05 was considered statistically significant.

**Results:**

A total of 70 ICU nurses participated in the study. No significant differences in alarm fatigue were found between nurses working more than eight days per month and those working fewer days (t(56) = 1.00, *p* = 0.32), and with more than one year of ICU experience and less experience, respectively (t(56) = 1.32, *p* = 0.19). Complete CAFQa data were available for 59 participants. The mean CAFQa score was (17.7 ± 5.5) points out of 36 points ((49 ± 15) %), indicating moderate to high alarm fatigue. Individual scores ranged from 5 (14%) to 28 (78%). The average self-reported alarm fatigue was (47 ± 22) %.

**Conclusions:**

Alarm fatigue is present among ICU nurses in moderate to high levels, but no significant differences were observed based on working hours or professional experience. Since alarm fatigue may have grave repercussions for nurses and patients, recognition and implementation of effective strategies to minimise it are crucial.

**Trial registration:**

Clinical trial number: Not applicable.

## Background

The overload of clinical alarms is among the top 10 health technology hazards, according to the Health Technology Hazards Executive Brief by the Emergency Care Research Institute (ECRI) in 2020 [1]. Alarm fatigue occurs when health care workers are exposed to an overwhelming number of alarms, which can lead to desensitization or improper use of alarm settings, resulting in reduced patient safety due to an insufficient or delayed response [2, 3]. ICU staff are unavoidably exposed to a large number of alarms generated by patient monitoring and other medical devices during their daily work. Alarm fatigue has been a concern for over a decade [4]. The number of medical devices that generate alarms has increased significantly, and nursing staff may get overwhelmed by the constant exposure and, consequently, experience alarm fatigue [1, 5].

The purpose of alarms is to increase patient safety by shortening the reaction time between the occurrence of a problem, for instance, potentially dangerous changes in vital parameters or failures of equipment, and the required response or intervention by attracting attention through acoustic or visual signals [4, 6]. However, alarms can also be triggered by artifacts or mismeasurements. Multiple studies over the last decades have shown that most alarms are false or not clinically relevant, indicating that 74 to 99% of all alarms did not require attention or clinical intervention [7–12]. A more recent study conducted by Andrade-Méndez et al. also showed that the clinical relevance of most alarms was medium to low [13]. Since alarm fatigue may adversely impact patients and ICU personnel, assessing the alarm fatigue level is essential to provide safety and investigate effective coping strategies [14]. Even though alarm fatigue has already been identified as a problem in ICUs years ago, and there have been attempts to develop a measuring tool, a gold standard has not been established yet [2, 15].

We used the Charité Alarm Fatigue Questionnaire (CAFQa) developed by Wunderlich et al. to assess alarm fatigue in ICU nurses [2]. To our knowledge, studies evaluating alarm fatigue among ICU nurses in Germany using the CAFQa are limited. This study provides a single-centre application example.

The research question addressed the extent to which alarm fatigue is present among ICU nurses in a German University Hospital and whether working hours and ICU experience have an impact on alarm fatigue.

## Methods

### Ethics approval

This analysis is covered by the approval of the ethics committee of the School of Medicine and Health, Technical University of Munich (Ismaninger Straße 22, 81675 Munich, Chairperson Prof. Dr. G. Schmidt, Reference 264/21S) as well as the hospital’s workers council. Participation in this study was entirely voluntary and anonymous. No separate informed consent procedure was necessary. Consent was implied by the return of the questionnaire. The study was conducted in accordance with the Declaration of Helsinki.

### Setting and sample

The study was conducted between January 2024 and March 2024, after the COVID-19 pandemic. It was a cross-sectional study to assess alarm fatigue in ICU personnel working in the TUM University Hospital Rechts der Isar in Munich, Germany. The ICUs had a surgical, medical, or toxicological focus. Participation was voluntary and there was no incentive or payment for participation. The inclusion criteria were the participants’ consent to participate in the study by return of the questionnaire and their affiliation to a participating ICU. Our research included nurses from five TUM University Hospital Rechts der Isar ICUs via an online survey. The data was collected through the platform LamaPoll [16], which allowed participants to access the survey anonymously through a direct link.

### Data collection

The CAFQa was used to assess the alarm fatigue of ICU nurses. Additionally, 18 items which were used during the development of the CAFQa, regarding alarm frequency, alarm perception, assignments to alarms, handling of alarms and workflow were collected. Each item of the questionnaire is scored on a five-point Likert scale (4 = “I very much agree”, 3 = “I agree”, 2 = “I agree in part”, 1 = “I do not agree”, 0 = “I do not agree at all”). Additionally, the participants were asked to rate their alarm fatigue on a visual analogue scale from 0 to 100%. It was possible to abstain from answering specific questions. Above that, the participants were asked to indicate whether they had “more than one year” or “one year or less” of professional experience, and whether they worked “more than eight days” or “eight days or less” per month on average in the ICU. To protect the anonymity of participants, we chose not to collect data on age, gender, and affiliated ICU. In case of a small sample size, which was possible as our study was conducted in a single centre, collecting such information could increase the risk of participant re-identification. We decided to use a web-based survey tool to ensure that transcription errors were ruled out and to enable faster analysis of the responses. Above that, web-based surveys allow participants to complete the questionnaire at a time and location of their choosing, thereby accommodating individual schedules and potentially reducing time pressure or situational stress that could influence their responses. LamaPoll adheres to German data protection regulations and fully supports anonymous surveys [16]. Although the quality of responses in web-based surveys has been questioned in the past, a recent study conducted by Clement et al. does not substantiate that self-administered web-based surveys encourage greater satisficing behaviour [17]. Furthermore, they found no indications that web-based surveys were more cognitively burdensome or distracting [17]. A potential limitation could be that web-based surveys require a certain amount of technological literacy. Since the study was carried out in a University Hospital, with our ICUs being highly digitised, one can assume that our ICU personnel are comfortable with web-based surveys. The estimated time to complete the questionnaire was approximately eight minutes.

### The CAFQa alarm fatigue questionnaire

The CAFQa was developed by Wunderlich et al. in 2023 and is aimed at nurses and physicians [2]. The questionnaire consists of 9 items covering the psychophysiological effects of alarms and the staff’s coping strategies in working with alarms. It is scored based on a 5-point Likert scale from 0 (“I do not agree at all”) to 4 (“I very much agree”). Items 6, 7, 8, and 9 have a negative valence and, therefore, are reverse-scored, i.e., 0 (“I very much agree”) to 4 (“I do not agree at all”). The range of scores in the CAFQa alarm fatigue questionnaire was from 0 (no alarm fatigue at all) to 36 (extreme alarm fatigue). A higher score indicated higher alarm fatigue. The developers suggested converting the score into a percentage to describe the level of alarm fatigue more intuitively.

To date, there is no established gold standard for measuring alarm fatigue [2]. Thus, we decided to use the CAFQa in our study since it fulfilled the following conditions: First, the CAFQa is transparent about its validated language, which in this case is German [2]. The alarm fatigue questionnaire developed by Torabizadeh et al. did not specify its original language or the translation process [15]. To avoid low-quality translations or second-level translations of non-validated first-level translations, which may result in a flawed interpretation, we chose to use the CAFQa. Above that, the development of the CAFQa adhered to the best practices of scale construction, which is not the case for other frequently used alarm fatigue questionnaires [2]. Alarm fatigue is a phenomenon that not only affects nurses [3]. Although we did not include physicians in our analysis, the CAFQa targets them as well, and we believe that a standardized measuring tool should all medical professions in the ICU. Two independent studies have substantiated the construct validity of the CAFQa alarm fatigue questionnaire. It is a reliable instrument for measuring alarm fatigue in nurses and physicians [2, 18].

### Statistical methods

The collected data was analysed using Microsoft Excel and OriginPro, Version 2023 (OriginLab Corporation, Northampton, MA, USA) [19]. Qualitative variables, like ICU experience (“one year or less” or “more than one year”) and monthly workdays (“8 days or less” or “more than 8 days”), are expressed as percentages and frequencies. Quantitative variables are presented as mean values and standard deviations. The Shapiro-Wilk test was used for confirmation of the normal distribution of the data. A two-sample t-test was used to assess statistical significance. The correlation between the participants’ CAFQa score and their self-evaluation of alarm fatigue was analysed using Pearson’s correlation coefficient. The threshold for statistical significance was set at *p* < 0.05. The internal consistency of each subscale was assessed using Cronbach’s α. Values of 0.70 or higher were considered indicative of acceptable reliability. Graphs were plotted by OriginPro, Version 2023 (OriginLab Corporation, Northampton, MA, USA) [19].

## Results

Between January 2024 and March 2024, 70 nurses returned the questionnaire and therefore participated in the study and were included in the analysis. Overall, 67 participants (96%) provided answers to 24 items or more, while 3 participants (4%) only answered 21–23 items.

A complete dataset of all nine CAFQa items is available for 59 participants; thus, the response rate regarding the CAFQa is 84%. 47 participants (67%) provided a complete dataset of all 27 questionnaire items (i.e. 9 CAFQa items and 18 additional items, as described in the Methods section). 69 participants responded to the questions regarding their monthly workdays (“8 days or less” or “more than 8 days”) and professional experience in ICUs (“one year or less” or “more than one year”). The study population is presented in Table 1. Most participants had more than one year of experience working in an ICU (*n* = 61, 87%) and are working more than eight days on average per month (*n* = 59, 84%).

**Table 1:** Study population

		*n*	%
Overall participants		70	100
all CAFQa items completed		59	84
all questionnaire items completed (CAFQa and additional items)		47	67
at least 24 items completed		67	96
Professional experience	1 year or less	8	11
	more than 1 year	61	87
	not specified	1	1
Workdays per month	8 days or less	10	14
	more than 8 days	59	84
	not specified	1	1

Table legend. Description of the study population. All participants (*n* = 70) were included in the analysis

The results are presented in the following subcategories:

1. Evaluation of the CAFQa alarm fatigue questionnaire,
2. Alarm fatigue level self-evaluation,
3. Alarm frequency,
4. Alarm perception and identification of the source,
5. Reaction and assignments to alarms,
6. Procedural instructions and handling of alarms,
7. Alarm limits, effects of alarms on participants, and interruption of workflow

### Evaluation of the CAFQa

**Fig. 1:**
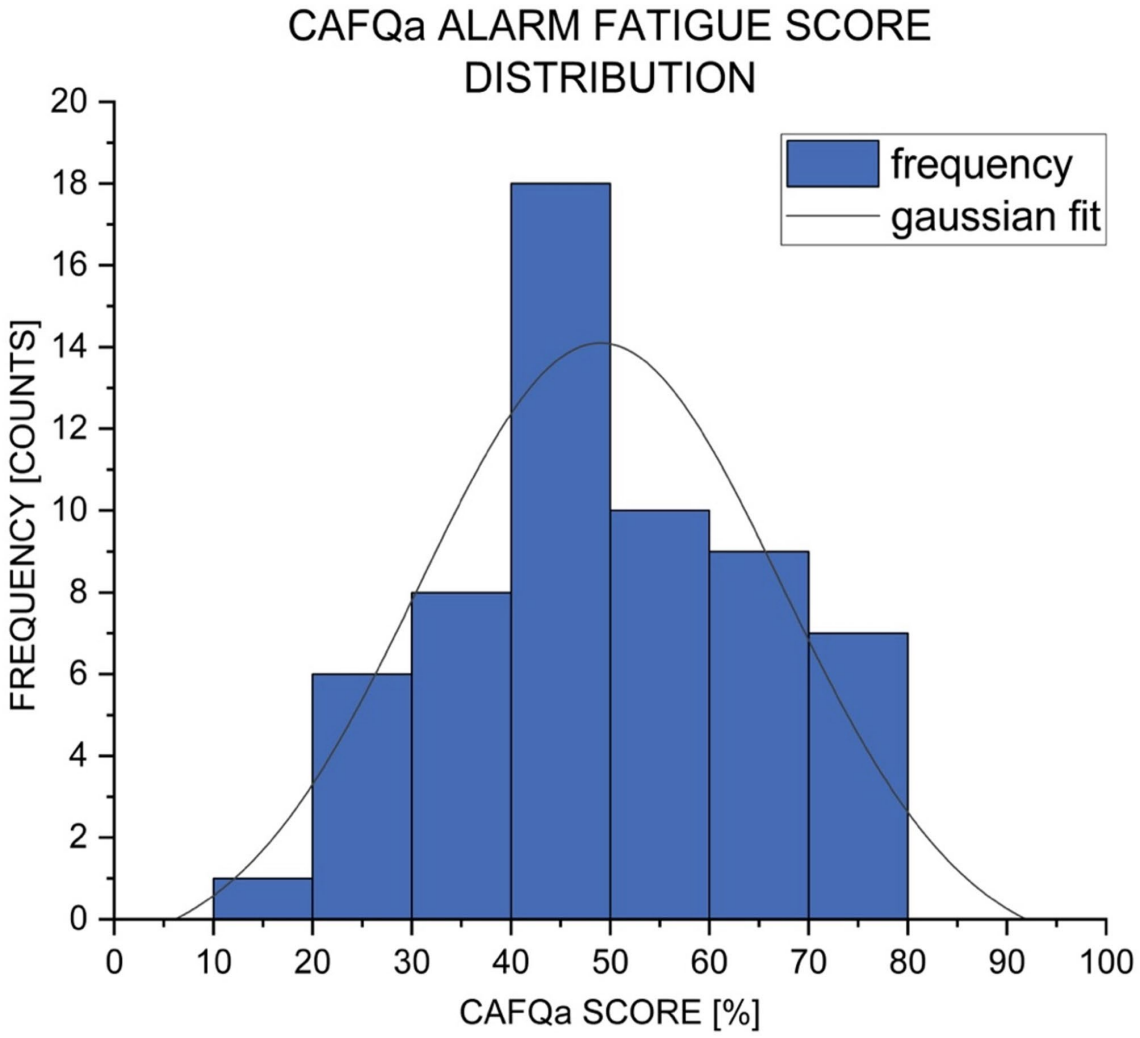
CAFQa alarm fatigue score distribution. Distribution of the participants’ CAFQa scores (*n* = 59). Numbers are presented as percentage (CAFQa Score) and counts (Frequency). Blue bars represent the observed frequency, while the black line indicates the corresponding Gaussian fit

The overall mean score was (17.7 ± 5.5) points or (49 ± 15) %, which indicates a moderate to high level of alarm fatigue. The lowest score obtained by a participant was 5 points (14%) while the highest was 28 points (78%). Figure 1 shows the distribution of the participants’ CAFQa scores. ICU nurses working more than eight days per month (*n* = 49) had a higher mean score than nurses working less (*n* = 9). ICU nurses with more than one year of ICU experience (*n* = 52) had a higher mean score than nurses with less experience (*n* = 6). A two-sample t-test indicated no significant difference between different amounts of working hours, t(56) = 1.00, *p* = 0.32, or ICU experience, t(56) = 1.32, *p* = 0.19. Internal consistency reliability was calculated for both subscales. The first subscale (5 items) showed acceptable reliability (Cronbach’s α = 0.78), whereas the second subscale (4 items) demonstrated lower reliability (Cronbach’s α = 0.53). A more detailed analysis of different groups of participants and the CAFQa items can be found in Tables 2 and 3.

**Table 2:** Alarm fatigue level self-evaluation vs. CAFQa score

		Alarm fatigue (%) Self-evaluation	Alarm fatigue (%) CAFQa	CAFQa score (points)	*p*-value
Overall		47 ± 22	49 ± 15	17.7 ± 5.5	
Workdays per month	8 days or less	49 ± 19	45 ± 10	16.1 ± 3.5	0.32
	more than 8 days	47 ± 22	50 ± 16	18.1 ± 5.8	
Professional experience	1 year or less	44 ± 22	42 ± 13	15.0 ± 4.7	0.19
	more than 1 year	48 ± 22	50 ± 15	18.1 ± 5.6	

Table legend. Mean and standard deviation of nurses’ alarm fatigue levels for various amounts of working hours and ICU experience. Comparison of self-evaluation in percent and CAFQa score in percent and points

**Table 3:** CAFQa items in detail

	Possible range	Min – Max	Mean (SD)
Overall	0–36	5–28	17.7 ± 5.5
1. With too many alarms on my ward, my work performance and motivation decrease.			1.9 ± 1.2
2. Too many alarms trigger physical symptoms for me, e.g., nervousness, headaches, sleep disturbances.			1.9 ± 1.5
3. Alarms reduce my concentration and attention.			2.1 ± 1.3
4. My or neighbouring patients’ alarms or crisis alarms frequently interrupt my workflow.			2.3 ± 1.0
5. There are situations when alarms confuse me.			1.8 ± 1.3
6. * In my ward, a procedural instruction on how to deal with alarms is regularly updated and shared with all staff.			2.9 ± 1.1
7. * Responsible personnel respond quickly and appropriately to alarms.			1.8 ± 0.9
8. * The audible and visual monitor alarms used on my ward floor and cockpit allow me to clearly assign patient, unit, and urgency.			1.7 ± 0.9
9. * Alarm limits are regularly adjusted based on patients’ clinical symptoms (e.g., blood pressure limits for conditions after bypass surgery).			1.4 ± 1.1

Table legend. The items of the CAFQa alarm fatigue questionnaire in detail. Each item is scored based on a 5-point Likert scale from 0 (“I do not agree at all”) to 4 (“I very much agree”). Items marked with an asterisk (*) were reverse-scored to improve comparability of the results

### Alarm fatigue level and self-evaluation

In addition to the 27 questions, the participants were asked to estimate their level of alarm fatigue on a visual analogue scale ranging from 0 to 100%. 65 participants answered this question.

**Fig. 2:**
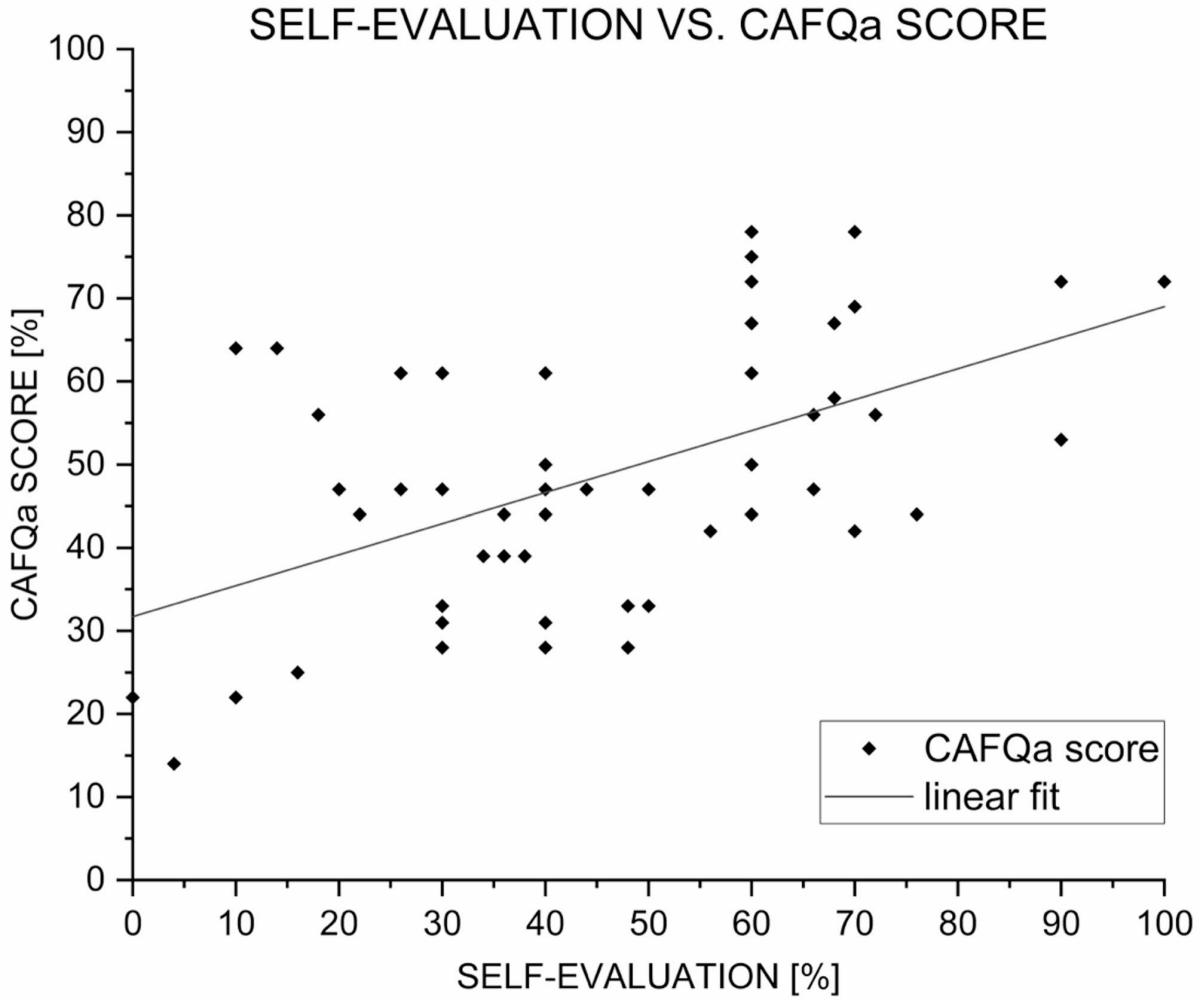
Self-evaluation vs. CAFQa score. Correlation between self-evaluated alarm fatigue and CAFQa scores. Scatterplot illustrating the relationship between self-reported alarm fatigue (%) and CAFQa scores (%) among ICU nurses (*n* = 59). Each point represents an individual participant. The solid line indicates the linear regression fit. A significant positive correlation was observed (Pearson’s *r* = 0.52; *p* < 0.0001)

The mean value was (47 ± 22) %, with the lowest percentage given by a participant being 0% and the highest being 100%. The level chosen most by participants (*n* = 11, 17%) was 60%. The CAFQa score and the corresponding percentage of each participant (*n* = 55) have been compared to the self-evaluation of their alarm fatigue. This can be found in Fig. 2. A Pearson correlation analysis revealed a significant positive correlation (*r* = 0.52; *p* < 0.0001).

### Alarm frequency

66% indicated “Alarms are too frequent on my ward”, with 30% agreeing and 36% strongly agreeing. Nobody disagreed strongly. The mean score on this item was (3.0 ± 0.9) points.

63% (26% agreeing strongly and 37% agreeing) stated that alarms were frequently triggered without any danger to patients. The mean score was (2.8 ± 0.9) points. When asked about critical or “red” alarms being too frequent, fewer participants (40%) agreed or strongly agreed. The mean score here was (2.1 ± 1.3) points.

### Alarm perception and identification of the source

When identifying the device at the patient’s bedside that triggers an alarm, most participants (73%) stated they do not have difficulties. More precisely, 23% disagreed and 50% disagreed strongly with the statement “It is difficult to identify the medical device at the patient’s bedside which triggers a relevant alarm.” The mean was (0.9 ± 1.2) points. However, correctly identifying patient, source, and urgency was more difficult when only acoustic and visual alarm signals were available, for example, on the ward floor or in the cockpit. In this case, only 20% stated they could make an assignment, with 4% agreeing strongly and 16% agreeing. Most participants (41%) agreed in part. The mean was (2.2 ± 1.0) points.

### Reactions and assignment to alarms

Participants were asked to provide their opinion on whether the responsible personnel react quickly and appropriately to alarms. 61% agreed to the statement “On my ward, only my occupational group responds to alarms”. The mean value was (2.8 ± 1.2) points. In general, 31% of the participants stated that responsible personnel quickly and appropriately addressed alarms.

### Procedural instructions and handling of alarms

11% stated that alarm management is addressed regularly on their ward. 50% of the participants discuss further procedures with their colleagues when alarms are recurring too frequently. 11% receive procedural instructions, which were updated and shared regularly on their ward regarding alarms.

### Alarm limits, effects of alarms on participants and interruption of workflow

The majority stated they always check alarm limits when starting their shift (83% with 79% agreeing strongly and 4% agreeing). The mean was (3.6 ± 0.9) points. The distribution of the answers to whether too many alarms trigger physical symptoms was almost flat, with 17% agreeing strongly, 20% agreeing, 16% agreeing in part, 26% disagreeing, and 21% disagreeing strongly with the statement. The mean was (1.9 ± 1.4) points. 13% stated that they did not feel interrupted in their workflow by patients’ alarms or crisis alarms. 17% did not feel prevented from carrying out other tasks like drug administration or patient care. 56% indicated that they were at least sometimes confused by alarms.

## Discussion

The aims of our study were to assess the level of alarm fatigue among intensive care nurses after the COVID-19 pandemic in Germany and to investigate differences between various working hours and years of professional experience. We were able to show that intensive care nurses suffer from moderate to high alarm fatigue, which is reflected in a mean CAFQa score of (17.7 ± 5.5) points (out of 36 points) and a self-rated alarm fatigue of (47 ± 22) %. These results are consistent with previous studies conducted both before and during the COVID-19 pandemic and confirm alarm fatigue among ICU nursing staff as an ongoing challenge in intensive care [20–23]. However, no significant differences were observed based on working hours or professional experience. The lower reliability of the second subscale is consistent with the original validation studies of the CAFQa, which reported Cronbach’s α values of 0.49, 0.55 and 0.57 [2, 18]. Higher CAFQa scores were associated with greater self-reported alarm fatigue.

### CAFQa alarm fatigue questionnaire score and alarm fatigue levels

ICU nursing staff, who are highly exposed to alarms since they devote significant time to patient care and monitoring, are particularly vulnerable to developing alarm fatigue [14]. A systematic literature review in 2020 by Lewandowska et al. highlighted that ICU nurses feel overburdened having to deal with both work duties and being exposed to alarms continuously, disrupting their workflow and diminishing their trust in alarm systems [14]. During the COVID-19 pandemic, health care systems across the globe have been challenged by the rapid spread and increasing number of infections [24]. In 2022, Asadi et al. conducted a study investigating alarm fatigue and moral distress in ICU nurses during the COVID-19 pandemic in Iran using the nurses’ alarm fatigue questionnaire developed by Torabizadeh et al. in 2017 [15, 20]. Ding et al. used the same questionnaire in 2023 in China to investigate the relationship between alarm fatigue and burnout among critical care nurses [23]. The results of both studies showed moderate levels of alarm fatigue in nurses, confirming its worldwide significance [20, 23].

Our results showed that participants who worked eight days or fewer per month in the ICU had a lower mean score on the questionnaire than those who worked more. Nurses with more than one year of ICU experience scored higher on average than those with less experience, although these results were not statistically significant.

Research regarding the relationship between the nurses’ average working hours, experience, and alarm fatigue is limited. Storm et al. also found no significant relationship between the average hours of workload per week, the years of ICU experience, and the nurses’ alarm fatigue [25]. They hypothesized that alarm fatigue may be related to excessive weekly working hours and attributed the lack of statistical significance to the fact that the average nurses’ working hours and experience were within the normal range and that experienced nurses may have expertise in managing alarm fatigue [25]. Notably, the total number of working hours or years of ICU experience was not asked for in the questionnaire, which limits the ability to compare the rationales in our study.

A review by Michels et al. showed inconsistent findings regarding the relationship between professional factors (i.e. experience) or environmental factors (i.e. shift type, workload) and alarm fatigue [3]. A possible reason for the inconsistency regarding professional factor could be that experienced health care professionals might suffer from a prolonged exposure to alarms, but on the other hand might have developed effective coping strategies and are more experienced in managing alarms properly [3].

### Alarm frequency and noise

In our study, most participants agreed that alarms were too frequent on their ward. This aligns with the results of a review by Lewandowska et al. [14]. More than 20 years ago, the World Health Organization recommended a maximum sound level of 35 dB L_Aeq_ for hospital wards since noise may cause adverse health effects after prolonged exposure [26]. Over the years, several studies have shown that the noise levels in ICUs exceed the recommended limit [27–30]. Schmidt et al. conducted a study in Switzerland to assess the effects of ICU noise on health care workers and concluded that noise in ICUs may adversely affect well-being, stress levels, and performance of ICU personnel [31]. However, measurements of absolute noise levels were not part of our analysis and are not performed regularly in our ICUs.

### Effects of noise and alarm fatigue on ICU nurses and patients

Alarm fatigue is an important patient safety issue and is associated with negative effects on working conditions and work performance of ICU personnel [32]. It has been linked to psychological and physical symptoms such as burnout, fatigue, headaches, and sleep disturbances [23, 33, 34, 35]. In the present study, the distribution of the answers to whether too many alarms trigger physical symptoms, e.g., nervousness, headaches, and sleep disturbances, in participants was almost flat with a slight tendency towards “I disagree”. This indicates that alarm overload expresses itself with a high individual variability.

Our results showed that nurses often were confused by alarms, felt interrupted in their workflow and were prevented from carrying out drug administration or patient care. Gündoğan et al. found that increased alarm fatigue is associated with an increased tendency to make medical errors in terms of medication and transfusion application, fall prevention, material safety, infection prevention and communication [22]. High-frequency alarms constantly divert the nurses’ attention away from their current tasks and thus impair the correct execution of work processes [1, 22]. However, the nurses’ medical error rate was not assessed in our study, precluding any assessment of potential associations between error rates and the observed outcomes. Alarms also have negative effects on patients’ well-being. The noise exposure can lead to sleep disorders, stress, delirium and subsequently to a prolonged hospital stay [30, 36]. Nurses’ alarm fatigue and the need to divide their attention may impair patient care and thus lead to decreased patient safety and further negative effects [1].

### Procedural instructions

Procedural guidance (“In my ward, a procedural instruction on how to deal with alarms is regularly updated and shared with all staff”) was scored with (2.9 ± 1.1) out of 4 points, with only 11% of nurses receiving regular updates. Simultaneously, most participating nurses reported feeling interrupted in their workflow or prevented from carrying out drug administration or patient care due to false alarms.

To date, data concerning the effect of alarm management programs on alarm fatigue is very limited, and further research is needed [37]. Given the well-documented consequences of alarm fatigue on patient safety and staff performance, the perception and feelings of inadequate and infrequently updated procedural instructions underscore the need for systematic training in alarm management [37–40]. Such trainings should aim to operationalize standardized procedures and strengthen nurses’ competencies in dealing with alarm systems effectively [39].

### Implications for clinical practice

Although we did not directly assess the nurses’ medical error rate or the impacts on work processes, several approaches were made to address the potentially negative impacts of alarm fatigue. After the study, meetings with the ICU department heads were scheduled to discuss the results and possible solutions. During these discussions, the rather critical findings regarding practice guidelines and onboarding processes were also addressed, as participants indicated that they often receive little guidance in their daily routine on how to handle alarms. The manufacturers of several medical devices were contacted to eventually adjust the default factory settings, which had been modified to better fit clinical requirements. Furthermore, alarm fatigue has been added to the Advanced Training in Intensive Care curriculum to raise awareness and provide education. Several training courses regarding alarm management and alarm fatigue, such as “*One Minute Wonders*” and structured group training, have been offered. In small groups, participants brainstormed and discussed possible solutions to minimise alarm fatigue on their respective wards. Additionally, meetings were held with the practical nursing instructors to explore how these topics can be integrated into the onboarding and ongoing training of nursing. We intend to administer the CAFQa again in order to facilitate a longitudinal analysis and assess possible changes regarding the nurses’ alarm fatigue over time, expanding the survey to include physicians and other healthcare professionals.

### Limitations

This study has several limitations. It was a cross-sectional study with a relatively small sample size. All participants are affiliated with the same university hospital. Most participants have a similar amount of ICU experience and workdays per month. Due to the limited sample size and lack of variability in terms of working hours and experience, meaningful subgroup analyses – such as comparisons between different levels of experience and/or qualification, workloads, employment relationship (TUM University employee or temporary employee) or intensive care units (e.g. surgical vs. medical) – were not possible. Furthermore, we deliberately chose not to collect data on age, gender, highest level of education attained and specific ICU qualifications in order to protect the anonymity of participants. However, this also limits the ability to analyse possible relationships with demographic factors. The second subscale showed lower internal consistency, which is in line with the original validation studies [2, 18]. This suggests that the lower reliability is a known property of this subscale. Although the questionnaire was designed to be completed within approximately eight minutes, it is possible that some participants perceived it as too lengthy. Future studies might consider distributing a shorter version to further reduce the risk of survey fatigue and improve completion rates. Lastly, participation in this study was voluntary. Thus, it is likely that the participants are primarily staff members interested in or affected by the study topic. This may lead to bias. Larger longitudinal studies involving a more diverse study sample with different levels of professional expertise are needed. Generalization may be made with caution.

## Conclusion

Based on our findings, ICU nurses experience moderate to high levels of alarm fatigue with a significant correlation between self-assessment and CAFQa scores. While no significant differences were found regarding experience or workload, the perception of insufficient procedural instructions underscores the need for structured alarm management training. Given the potential negative impact on both health care workers and patients, it is crucial to recognise and implement effective strategies to minimise alarm fatigue. This study’s results will serve as the basis for improving our local processes and work environment.

## Data Availability

The datasets used and/or analysed during the current study are available from the corresponding author on reasonable request.

## Abbreviations

ICU: Intensive Care Unit
CAFQa: Charité Alarm Fatigue Questionnaire
